# Transcriptomic integration of D_4_R and MOR signaling in the rat caudate putamen

**DOI:** 10.1038/s41598-018-25604-4

**Published:** 2018-05-09

**Authors:** Alejandra Valderrama-Carvajal, Haritz Irizar, Belén Gago, Haritz Jiménez-Urbieta, Kjell Fuxe, María C. Rodríguez-Oroz, David Otaegui, Alicia Rivera

**Affiliations:** 1 Universidad de Málaga, Instituto de Investigación Biomédica, Facultad de Ciencias, Málaga, Spain; 2 Neuroscience Area, Biodonostia Institute, San Sebastián, Spain; 3 Centro de Investigación Biomédica en Red sobre Enfermedades Neurodegenerativas (CIBERNED), Madrid, Spain; 4 Present Address: Division of Psychiatry, University College London, London, England United Kingdom; 5 Neuroscience Department, Karolinska Institute, Stockholm, Sweden; 6 Neurology Department, Donostia University Hospital, San Sebastián, Spain; 7 Ikerbasque Foundation, Bilbao, Spain; 8 Present Address: Universidad de Málaga, Instituto de Investigación Biomédica, Facultad de Medicina, Málaga, Spain

## Abstract

Morphine binding to opioid receptors, mainly to μ opioid receptor (MOR), induces alterations in intracellular pathways essential to the initial development of addiction. The activation of the dopamine D_4_ receptor (D_4_R), which is expressed in the caudate putamen (CPu), mainly counteracts morphine-induced alterations in several molecular networks. These involve transcription factors, adaptive changes of MOR signaling, activation of the nigrostriatal dopamine pathway and behavioural effects, underlining functional D_4_R/MOR interactions. To shed light on the molecular mechanisms implicated, we evaluated the transcriptome alterations following acute administration of morphine and/or PD168,077 (D_4_R agonist) using whole-genome microarrays and a linear regression-based differential expression analysis. The results highlight the development of a unique transcriptional signature following the co-administration of both drugs that reflects a countereffect of PD168,077 on morphine effects. A KEGG pathway enrichment analysis using GSEA identified 3 pathways enriched positively in morphine vs control and negatively in morphine + PD168,077 vs morphine (Ribosome, Complement and Coagulation Cascades, Systemic Lupus Erythematosus) and 3 pathways with the opposite enrichment pattern (Alzheimer’s Disease, Neuroactive Ligand Receptor Interaction, Oxidative Phosphorilation). This work supports the massive D_4_R/MOR functional integration at the CPu and provides a gateway to further studies on the use of D_4_R drugs to modulate morphine-induced effects.

## Introduction

Morphine is a potent analgesic drug prescribed to relieve moderate to severe pain. It is well known that acute and prolonged exposure to morphine induces various molecular and cellular alterations in neurons of multiple brain areas, which may persist after use cessation and lead to addiction and relapse^1^. Morphine induces its effects at different levels such as receptors, intracellular signalling pathways, the expression pattern of transcription factors (i.e. Fos and CREB families), morphological features of neurons and their connectivity, which may be interrelated^2–7^. Many of these neuroadaptations are directly related to changes in dopamine release in the nucleus accumbens (NAc) and the caudate putamen (CPu), areas regulated by mesencephalic dopaminergic neurons^8^, which modulate reward, motor function and habit learning^9^.

It was previously described that the agonist of the dopamine D_4_ receptors (D_4_R) PD168,077 modulates, mostly through a blocking effect, the morphine-induced expression of transcription factors of the Fos and CREB families in the CPu^2,3^, as well as the sensitization of the μ opioid receptor (MOR)^3,10^. The ability of D_4_R activation to maintain basal dopamine release in the CPu during morphine exposure could be one of the underlying mechanisms for the counteractive effects of D_4_R^11^. Thus, a functional interaction between D_4_R and MOR has been suggested^11,12^ since both receptors are co-expressed in GABAergic neurons of the CPu and SNr^13–16^, which, inter alia, control the activity of dopaminergic neurons^17,18^. In this work we employed the polymerase chain reaction (PCR) technique to reconfirm and support that the D_4_R mRNA is expressed in the CPu.

Interestingly, the D_4_R agonist PD168,077 does not modify the analgesic properties of morphine^11^, opening up the possibility of a new pharmacological strategy for pain treatment by avoiding its addictive effects. An improved understanding of how D_4_R activation prevents the molecular changes induced by morphine could provide crucial knowledge to implement this therapeutic approach.

In this study, we present the results of a whole genome gene expression analysis aimed at elucidating the transcriptomic alterations involved in the acute D_4_R/MOR interaction. In a hypothesis free approach, we have generated dorsal striatal profiles of deregulated genes and cellular pathways after the administration of either morphine, PD168,077 or their combination in rats.

## Results

### Expression of D_4_R mRNA in the CPu

**Figure 1:**
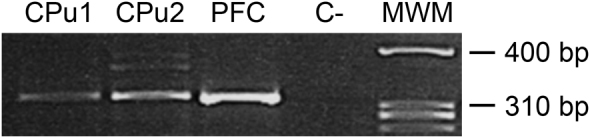
Dopamine D_4_R mRNA expression in the caudate putamen. Expression of transcripts encoding the dopamine D_4_R was analyzed regionally in the caudate putamen (CPu) from two pools (n = 3 each) and prefrontal cortex (PFC) from one pool (n = 3) of control animals. No signal was detected in the sample run without RNA sample (C−).

As shown in Fig. 1 and Supplementary Figure 1, the PCR analysis confirmed the expression of D_4_R mRNA in the CPu of the two different pools of control animals (n = 3 animals/pool). The D_4_R mRNA was also detected in the frontal cortex, being used as a positive control since the high expression of this dopaminergic receptor in this brain area^19,20^.

The sequencing of the PCR products corroborated their homology with D_4_R mRNA. Once sequenced, the complete identity of the amplicons as D_4_R coding region was ensured by sequence similarity (100%) with rat nucleotide database using Basic Local Alignment Search Tool (BLAST; National Center for Biotechnology Information, NCBI).

### Gene expression profiling

For this study, we assessed the transcriptional effects of morphine (10 mg/kg) and PD168,077 (1 mg/kg), alone or in combination, in the rat CPu. A time-course study (1 and 2 h) of gene expression alterations following acute administration of these drugs was performed to study the dynamics of early changes.

In order to investigate the effects of the injection on gene expression, a comparison between the control (C) groups, i.e. C1h and C2h, was performed. This analysis yielded a list of 21 genes (9 upregulated and 12 downregulated) that pass the |FC| > 2 and p-value < 0.05 (unadjusted) filter, with a maximum |FC| of 3.78 (Supplementary Table 1). As the deregulation of these genes could be a consequence of the injection of vehicle or false positives due to sample size limitations, they were considered as being spuriously altered (from now on “spurious genes”). Thus, these genes were excluded from the list of differentially expressed genes (DEGs) obtained in subsequent analyses with the aim of reducing the bias introduced by noise in the results.

Then, differential expression analyses were done by comparing each treatment group at each time point with its corresponding control group (i.e. morphine (M) 1 h with C1h, M2h with C2h, PD168,077 (PD) 1h with C1h, and so on) and the groups under one of the drug treatments with those treated with both drugs. Table 1 shows the lengths of the DEG lists obtained in the 6 comparisons describing the effect of each drug and the 4 comparisons performed to isolate the effect of the co-treatment. The number of upregulated and downregulated genes are presented both before and after the removal of the “spurious genes”. In total, 285 DEGs appear in any of the 10 comparisons, among which 222 were unique genes with Gene Symbols. Of these, 82 appear in one of the comparisons, 49 in 2 comparisons, 42 in 3 of them, 20 genes in 4, 13 genes in 5 comparisons, 8 genes in 6, 5 genes in 7 comparisons and, remarkably, 3 genes (Mfsd4, Neurod6 and Sstr1) appear as differentially expressed in 8 of the comparisons (Neurod6 has a FC of 2.2 in the C2h vs C1h, but its p-value is >0.05). A full list of DEGs is provided in Supplementary Table 2.

**Table 1:** Number of differentially expressed genes (DEGs) identified in the comparisons between groups.

DEGs (upregulated/downregulated)				
Comparison	All genes	Without “spurious genes”	Genes with symbol	Maximum |FC| value
C1h vs. C2h	9/12	0/0	8/6	3.8
M1h vs. C1h	31/0	24/0	26/0	6.1
M2h vs. C2h	17/3	16/0	12/0	40.7
PD1h vs. C1h	37/1	30/0	34/0	5.3
PD2h vs. C2h	2/1	2/0	0/0	3.5
MPD1h vs. C1h	180/3	172/2	154/1	17.7
MPD2h vs. C2h	133/7	125/7	109/5	28.6
MPD1h vs. PD1h	42/23	36/23	36/21	4.8
MPD2h vs. PD2h	61/14	59/14	49/11	21.8
MPD1h vs. M1h	54/3	46/2	48/2	5.7
MPD2h vs. M2h	138/12	130/12	116/10	9.2

First column includes all the DEGs; second column shows the result of filtering out the “spurious genes”; third column indicates the genes that have an assigned gene symbol; forth column display the maximum absolute Fold Change (|FC|) value found in each comparison. Abbreviations: C, control; M, morphine; PD, PD168,077; MPD, morphine + PD168,077.

Of note is the strong influence of the co-administration of morphine and the D_4_R agonist on gene expression. The number of DEGs is remarkably higher under the simultaneous effect of both drugs than under the effect of each drug separately at both time points (Table 1 and Supplementary Table 2). Besides, the effect of the treatments on gene expression seems to diminish with time as the length of the DEG list is reduced 2 h after the administration of the drugs (Table 1 and Supplementary Table 2).

### Identification of drug-related gene expression patterns

**Figure 2:**
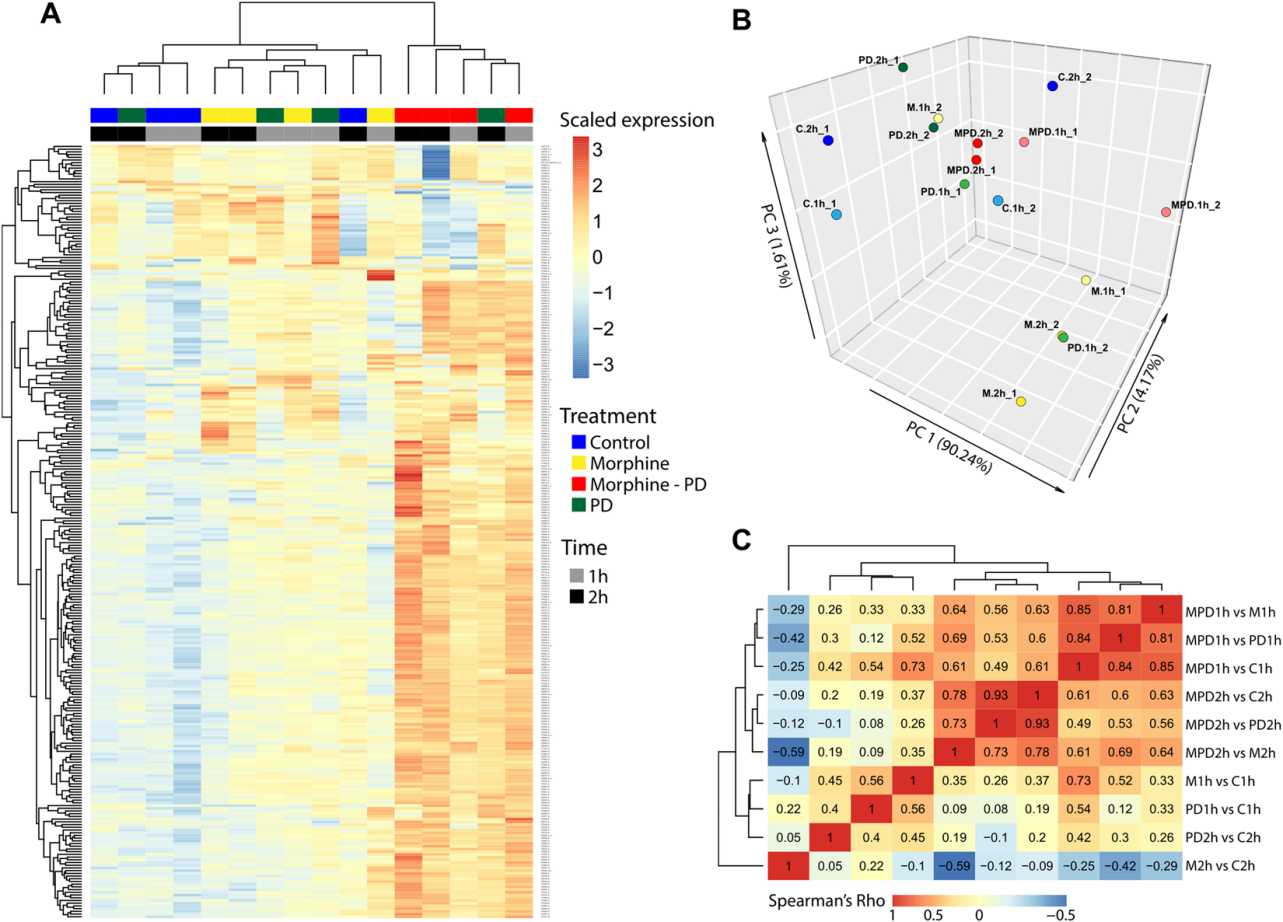
Treatment-induced alterations in gene expression. (**A**) Heatmap of the standardized expression of the 285 differentially expressed genes across the 16 samples. (**B**) Visualization of the 16 samples across the 3 most informative components of a principal component analysis performed on the raw expression values (log2-intensity) of the 285 DEGs. (**C**) Heatmap of the matrix of correlations between the fold-change profiles of the 285 DEGs in the 10 comparisons performed to explore the effect of the treatment regimes on gene expression.

In order to visualize the expression patterns of the 285 DEGs and explore the similarity/dissimilarity among the profiles, we standardized the expression data, performed a hierarchical biclustering of genes and samples and visualize the result as a heatmap (Fig. 2A). Although one of the PD-treated samples at 2 h appears clustered with the samples under the co-treatment regime, it is clear, overall, that the latter present a very distinct gene expression profile. This, along with the much larger number of DEGs observed under the co-treatment, suggests that an interaction has happened between the MOR and the D_4_R at the molecular level. We also performed a Principal Component Analysis (PCA) to further explore the relationship of the expression profiles for the DEGs among the samples. While the gene expression data needs to be standardized for the heatmap visualization to reveal distinct patterns across samples, we decided to use the normalized log2-intensity values directly for the PCA because a) all variables (the expression of the genes) are in the same units (log2-intensity values) and b) we considered that, by giving more weight to the highly expressed genes, we would obtain a result that reflects more faithfully the similarities between the gene expression profiles. Somehow contradictorily to what the heatmap shows, the visualization of the samples across the coordinates of the three most informative principal components reveals that, while the samples on morphine at 2 h appear on one edge of the plot, the ones under the co-treatment at 2 h lie much closer to the control samples (Fig. 2B). This suggests that the addition of the PD to the morphine counteracts the effects of the latter on gene expression at 2 h, at least to a certain extent. The fact that the fold-change profiles for those 285 DEGs observed in morphine vs control at 2 h and in morphine-PD vs morphine at 2 h are strongely negatively correlated (Spearman’s rho = −0.59 (p-value < 2.2e^−16^)) (Fig. 2C) further supports the idea of the addition of PD counteracting the effects of morphine at 2 h. In summary, the results indicate that there is interaction between the MOR and D_4_R at the molecular level and that the addition of the PD to morphine counteracts the effects of the latter on gene expression, at least to a certain extent.

### Pathways involved in the counteracting effect of PD

**Figure 3:**
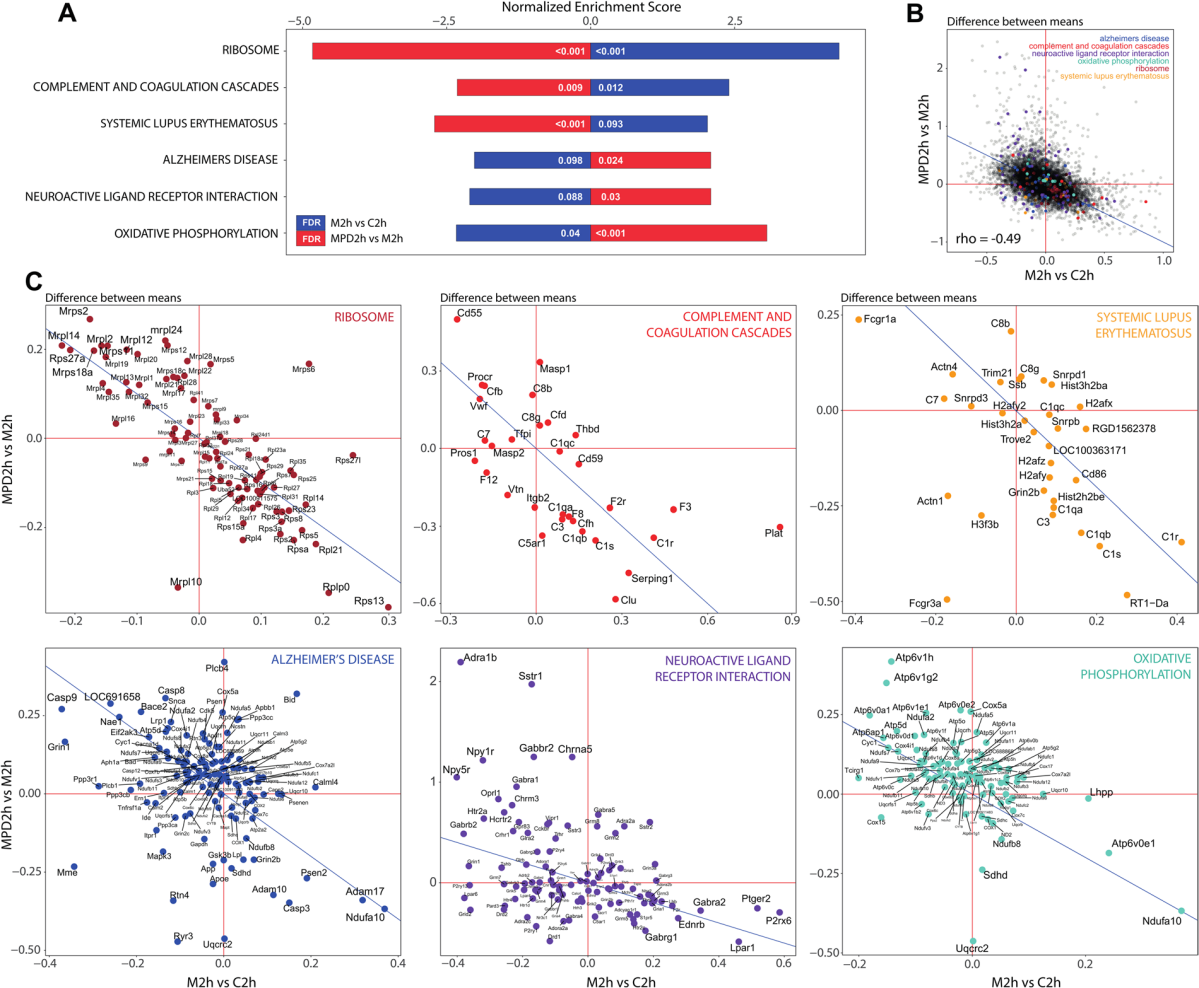
Pathways involved in the countereffect of PD. (**A**) Normalized Enrichment Scores and FDR values for the 6 KEGG pathways significantly enriched in the “M2h vs C2h” and “MPD2h vs M2h” comparisons in the opposite direction. (**B**) Scatterplot of the differences between the means in the “M2h vs C2h” and “MPD2h vs M2h” comparisons for all 19587 expressed genes. (**C**) Scatterplots of the difference between the means in the same two comparisons for the genes involved in the 6 enriched pathways. In all scatterplots, the blue line represents the “perfect countereffect” line (y = −x), that is, the line on which the genes would lie if the countereffect of the addition of PD would be perfect (for example ${\overline{x}}_{{\rm{M}}2{\rm{h}}}-{\overline{x}}_{{\rm{C}}2{\rm{h}}}=0.5$; ${\overline{x}}_{{\rm{MPD}}2{\rm{h}}}-{\overline{x}}_{{\rm{M}}2{\rm{h}}}=-\,0.5$).

We then explored what pathways could potentially be involved in the countereffect of the addition of PD at 2 hours revealed by the differential expression signatures. To that end, we performed a KEGG pathway enrichment analysis on M2h vs C2h and MPD2h vs M2h using GSEA^21,22^ and identified the pathways that are significantly enriched (FDR < 10%) on the opposite direction. Using the signed difference between the means as the parameter to rank the genes, the GSEA analysis revealed 3 pathways enriched positively in M2h vs C2h and negatively in MPD2h vs M2H (Ribosome, Complement and Coagulation Cascades and Systemic Lupus Erythematosus) and other 3 pathways with the opposite enrichment pattern (Alzheimer’s Disease, Neuroactive Ligand Receptor Interaction and Oxidative Phosphorilation) (Fig. 3A). A scatterplot of the differences between the means for the two comparisons (Fig. 3B) shows that the huge majority of the genes cluster around the perfect countereffect line (y = −x) with a strong negative correlation (Spearman’s rho = −0.49 (p-value < 2.2e^−16^)), suggesting that the countereffect of the addition of PD at 2 hours observed for the 285 differentially expressed genes is a global transcriptomic effect and validating the use of the differences between the means to rank the genes in the GSEA analysis. While the genes that belong to the enriched pathways do not form clusters in the overall scatterplot (Fig. 3B), the pathway-specific scatterplots (Fig. 3C) show that they are distributed around the countereffect line, specially those in the Ribosome, Complement and Coagulation Cascades and Systemic Lupus Erythematosus pathways. It is of note that the results point at a downregulation of mitochondrial activity produced by morphine that is recovered with the addition of PD as suggested by the downregulation in M2h vs C2h and upregulation in MPD2h vs M2h of the genes in the oxidative phosphorylation pathway and mitochondrial ribosomal protein (*Mrps* and *Mrpl*) genes.

### Validation of differentially regulated genes by quantitative RT-PCR

**Figure 4:**
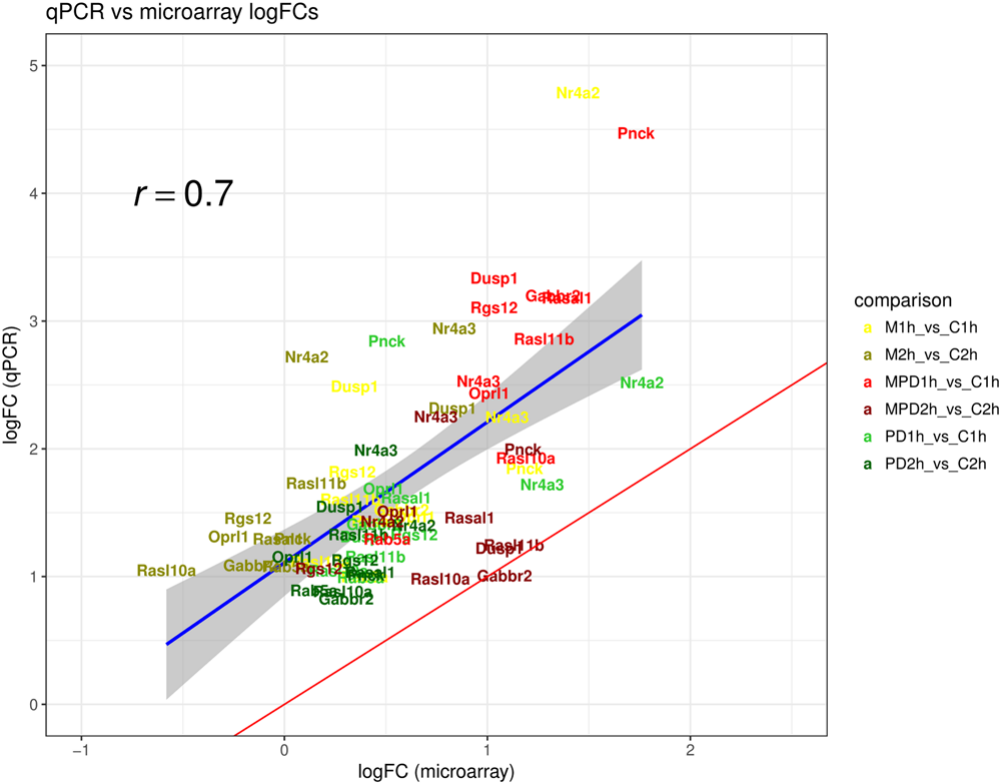
Results of the technical validation by qRT-PCR. Scatterplot of the logFC-s (log_2_Fold-change) obtained with microarrays and with qRT-PCR (qPCR) for the 11 genes included in the validation across 6 treatment vs control comparisons. The red line is the perfect validation line (x = y), that is, the line where genes would lie if their logFC-s were equal for both platforms. The blue line represents the association between the logFC profiles from each platform as given by a linear regression, with the shadow reflecting the 95% confidence interval.

A subset of 11 DEGs was selected to validate the treatment-induced regulation of gene expression observed at both timepoints in the microarray analysis by quantitative RT-PCR (qPCR). Among these genes, and based on gene ontology classification, five are involved in several cell signaling pathways (*Dusp1, Nr4a2, Nr4a3, Gabbr2, Oprl1*), another five are related to GTP binding and GTPase activity as well as to GTPase activator activity (*Rgs12, Rab5a, Rasal1, Rasl1*0*a, Rasl*1*1b*) and one is associated to calmodulin-binding and calmodulin-dependent protein kinase activity (*Pnck*). The qPCR results (Fig. 4) support the observations made on microarray data for both timepoints as the overall Pearson’s correlation value between log fold-changes (log2(FC)) is R = 0.7 (p-value < 5.74e^−11^).

## Discussion

Previous results have shown that the activation of D_4_R by its agonist PD168,077 selectively modifies morphine effects at different levels (gene expression, receptor characteristics, pathways, behavior, etc)^2,3,10,11^. In the present study, and in order to expand our knowledge of this phenomenon, a screening of the entire transcriptome using microarrays was employed to search for global gene expression alterations that occur in response to the acute administration of morphine and/or PD168,077.

The results show that the co-administration of both drugs induces a massive transcriptional regulation of genes in the rat CPu, probably due to the D_4_R-MOR interaction as we have previously demostrated^2,10,11,23^. This interaction seems to be based on the expression of both receptors in the striatum^13,14,16^. Although the presence of the D_4_R protein has already been described in the CPu^16^, we confirm the expression of D_4_R mRNA in the striatum in the present work.

Several observations indicate that the pattern of deregulation observed in the co-treatment cannot be explained by the aggregation of the alterations provoked by the single drug regimes, supporting the idea of an interaction between MOR and D_4_R at the molecular level. On one hand, there is a sizeable set of genes not shown to be altered in the single-treatment groups that are deregulated under the co-treatment regime. Besides, the heatmap of differentially expressed genes reveals a distinct, clearly distinguishable expression pattern for the MPD group. It likely involves allosteric receptor-receptor interactions in D_4_R-MOR heteroreceptor complexes in the CPu^11^. Besides, these results support the idea that the CPu have a the relevant role during the initial intoxication/binge stage of the addiction cycle mainly in drug habit acquisition, to the transition to compulsive drug- seeking^24–26^.

As a proof of concept, we checked the expression of some genes known to be linked to morphine effects. The co-administration of morphine and PD168,077 produced changes in genes involved in different G protein-coupled receptor signaling pathways or in their regulation. Among them, altered expression has been observed for *Dusp1* and *Gna14*, which regulate the mitogen-activated protein kinase (MAPK) pathway^27,28^, and for *Plcxd*2 and *Pnck*, which encode for proteins related to the phospholipase C pathway^29^. Considering that both MOR and D_4_R are G protein-coupled receptors (GPCRs) and that we have observed a deregulation of genes involved in GPCR signalling, our results suggest that a functional interaction between MOR and D_4_R could exist that alters the phosphorylation of multiple effectors through the regulation of both the phospholipase C and MAPK pathways. In fact, one of those effectors is the cAMP response element-binding protein (CREB), whose phosphorylated form is increased in the striatum after the acute co-treatment of morphine and PD168,077^2^. According to our results, the G-protein and cAMP mediated signaling and nucleotide second messenger pathways are altered by the co-treatment, too. Although these networks have also been shown to be linked to the morphine treatment^30^, in our data they are affected only by the cotreatment.

The brain-derived neurotrophic factor (BDNF) has been suggested to be required for the growth of striatal neurons and their dendritic complexity and spine density^31^, effect probably mediated by its binding to the receptor TRKB. It has also been pointed out as a negative modulator of morphine effects^32^, at least in the ventral tegmental area (VTA)-NAc signalling. Although morphine alone did no alter the expression of the gene encoding BNDF (*bdnf*), other drugs of abuse such as alcohol and cocaine can alter its levels in the striatum^33,34^. We have observed that the D_4_R agonist PD168,077 and the co-treatment induce it, so the D_4_R-mediated over-expression of this neurotrophic factor may contribute to the regulation of the effects of morphine on the substantia nigra-CPu dopaminergic signaling^11^. The administration of morphine and PD168,077 also modulates other genes related to neurite outgrowth (*Homer1*, *Pfn2* and *Slitrk1*), which may indicate a mechanism underlying the D_4_R modulation of morphine-induced effects on neuronal structure. In fact, the agonist of D_4_R prevents morphine-induced alterations of SNc dopamine neuronal morphology^11^. Previous studies strongly demonstrated the role of D_4_R in modulating, mainly through a counteracting effect, molecular and cellular as well as rewarding and physical withdrawal effects of morphine after acute and subchronic administration^2,10,11^.

We pointed out that a potential countereffect of PD168,077 was revealed by the PCA and the heatmap of correlations between fold-changes of the comparisons. In the analysis of the KEGG pathways, it is notorious that genes involved in both oxidative phosphorylation (e.g., *Ndufa*, NADH dehydrogenase; *Cyc1*, cytochrome c1) and ribosome pathways (e.g. *Mrps* and *Mrpl*, mitochondrial ribosomal proteins) were downregulated after acute morphine treatment but upregulated after the adition of PD168,077 to morphine. The downregulation of genes involved in mitochondrial oxidation by morphine has been previously observed^35,36^ and linked to its antioxidant and protective effects (Gulcin *et al*., 2004). Regarding the neuroactive ligand and receptor interaction pathway, morphine altered not only dopaminergic and opioid neurotransmission^2,3,8,11^ but also other neurotransmitter systems as GABA (e.g. *Gabbr2, Gabrb2, Gabra1*), NPY (e.g. Npy1r, Npy5r) and adrenaline (e.g. Adra1b) and their expression are upregulated after morphine + PD168,077 administration.

In conclusion, our data show that the acute co-treatment with morphine and the D_4_R agonist PD168,077 has a dramatic effect on the expression of the transcriptome in the CPu. This effect presents a unique signature that includes genes belonging to crucial networks for drugs producing addiction. Therefore, these results represent an important advance in the understanding of the mechanisms by which D_4_R counteracts the early effects of morphine, providing molecular evidence to the D_4_R/MOR interaction. This work open a gateway to develop further studies to deep into the networks involved in the interaction mechanisms after both acute and chronic administration, focussing on rewarding and addictive effects and the development of novel therapies for pain treatment.

## Methods

### Animals

Male Sprague-Dawley rats (Harlan Laboratories Inc., UK) (n = 48) weighing 225 to 250 g were used in all sets of experiments. Animals were maintained on a 12 hour light/dark cycle in temperature- and humidity-controlled conditions (22 ± 2 °C, 55–60%) with free access to water and food. All experimental procedures were carried out in accordance with the guidelines of the European Communities Council (2010/63/EU) as well as the Spanish Government (RD 53/2013) Directives and were approved by the Ethics Committee for Animal Testing at the Biodonostia Institute (Spain).

### Drug treatments

Animals received a single intraperitoneal injection (i.p.) of either morphine sulphate (10 mg/kg; supplied by Ministerio de Sanidad, Política Social y Consumo (Spanish Government)), PD168,077 (1 mg/kg; Tocris Bioscience, UK) or both drugs, which were dissolved in 2% DMSO within 0.9% NaCl (vehicle). Animals were sacrificed by decapitation at 1 h or 2 h after treatment (n = 6 per group) (Table 2).

**Table 2:** Experimental groups of the study.

Time point (h)	Treatment	Group	Dose (mg/kg)
1h	vehicle	C1h	
	morphine	M1h	10 mg/kg
	PD168,077	PD1h	1 mg/kg
	morphine + PD168,077	MPD1h	10 mg/kg + 1 mg/kg
2 h	vehicle	C2h	
	morphine	M2h	10 mg/kg
	PD168,077	PD2h	1 mg/kg
	morphine + PD168,077	MPD2h	10 mg/kg + 1 mg/kg

Groups were based on the drug treatment and time of sample extraction (n = 6 per group). RNA from three animals were pooled to perform the analysis (n = 2 pools per experimental group).

### Tissue preparation and RNA extraction

Brains were removed and a punch from the left hemisphere CPu and frontal cortex was taken, rapidly placed in 500 μl of RNAlater solution (Ambion, Life Technologies, Carlsbad, CA) and immediately frozen and stored at −80 °C to prevent RNA degradation.

Total RNA was isolated using Qiazol (Qiagen Science, Germantown, MD) and RNeasy Lipid Tissue Mini kit (Qiagen Science, Germantown, MD) according to instructions of the manufacturer. The concentration, purity and integrity of total RNA was determined spectrophotometrically by measuring the absorbance ratio at 260/280 nm (Nanodrop ND1000; Thermo Scientific, Waltham, MA) and using the RNA 6000 Nano LabChip kit with the Agilent Bioanalyser (Agilent Technologies, Santa Clara, CA). Only the samples with a 260/280 nm ratio ≥1.9 and no signs of degradation were used. Out of six RNA isolated samples per experimental group, two pools per treatment and time point were obtained (n = 3) (Table 2).

### PCR analysis of D_4_R mRNA

The polymerase chain reaction (PCR) was employed together with specific oligonucleotide primers and DNA sequencing techniques. CPu samples of the two pools of vehicle-injected animals and a sample from the frontal cortex of one pool of vehicle-injected animals were analysed. Based on rat D_4_R mRNA sequence (NM_012944.2), a set of primers was designed using the free online software Primer3 (http://primer3.sourceforge.net/) so intron-exon boundaries would not be crossed: (1) F 5′-TGCAGAAATTCAAGCCGTGG-3′ and R 5′-GCCAGGCTCACGATGAAGTA-3′. After reverse transcription, the PCR was performed in a 25-μl-reaction mixture in a Veriti 96 well thermal system (Thermo Fisher Scientific) with 45 cycles of 95 °C for 15 s, 60 °C for 30 s, 72 °C for 30 s, and a final elongation step of 72 °C for 10 minutes. The amplified PCR products were evaluated by 3% agarose gel electrophoresis. Once verified the specificity and the size of the fragment as expected *in silico* (≈260 bp), the rest of the PCR product was used for sequencing at the Genomics Platform of the Biodonostia Health Research Institute (San Sebastián, Spain) using the same forward primer employed for PCR amplification. Sequence quality was assured by free software BioEdit (http://www.mbio.ncsu.edu/BioEdit/bioedit.html), and alignment and homology search with rat exome was performed using BLAST (National Center for Biotechnology Information, NCBI) to determine and confirm the homology with D_4_R mRNA sequence.

### Microarray assay

Whole genome gene expression analysis was performed with the GeneChip Rat Genome 230 2.0 array (Affymetrix, Santa Clara, CA), which contains more than 28,000 rat genes, according to the instructions of the manufacturer. In brief, 150 ng of total RNA from each independent sample pool were transcribed to cDNA, amplified, and subjected to hybridization using the Affymetrix Fluidic Station 450 (Affymetrix, Santa Clara, CA) and the hybridization oven under standard conditions.

The probe-level signal data obtained from scanned chip images (GeneChip 7G Scanner; Affymetrix) were summarized and normalized using the Robust Multi-array Average (RMA) algorithm.

### Differential expression analysis

The differential expression analysis was done using the *limma* package^37^ in R. The multiple linear regressions were performed by fitting the following model:

1 $$Y={Y}_{0}+{{\rm{\beta }}}_{1}{X}_{1}+{{\rm{\beta }}}_{2}{X}_{2}+{{\rm{\beta }}}_{3}{X}_{3}+{{\rm{\beta }}}_{4}{X}_{1}{X}_{2}+{\rm{\varepsilon }}$$

where *y* is the normalized gene expression in log2 scale, *y*_*0*_ is the average gene expression in the reference group (time = 1 h, treatment = control), *x*_*1*_, *x*_*2*_ and *x*_3_ are the experimental time, the treatment regime and the microarray batch, β_1,_ β_2_ and β_3_ are the corresponding effect coefficients, β_4_ is the coefficient for the interaction between time and treatment and ε is the residual variance. For each comparison, the genes with a |FC| > 2 and a p-value < 0.05 (unadjusted) were considered to be significantly differentially expressed.

The rest of the analyses on the differentially expressed genes and the visualizations of the results were performed using several R packages. The heatmaps were generated with the *aheatmap* function of the *NMF* package^38^, the principal component analysis was done with the *PCA* function of the *FactorMineR* package^39^ and its results visualized with the *scatter3D* function of the *plot3D* package.

### Pathway enrichment analysis

The KEGG pathways’ enrichment analysis was done with the GSEA software^21,22^. The “c2.cp.kegg.v6.1.symbols.gmt” was selected as the database of gene-sets to compute enrichment for and 1000 gene-set-wise permutations were performed to generate the null distributions. Genes were ranked in descending order based on the signed difference between the classes and the enrichment scores were calculated using the classic statistic (unweighted). The corresponding microarray (Rat230_2.chip) was selected as background. The KEGG pathways with an FDR < 10% were considered to be differentially expressed. The column-plot showing the significantly enriched KEGG pathways and the corresponding scatterplots were generated using *ggplot2*^40^ (R package).

### Quantitative PCR analysis

From the differentially expressed gene-list, 11 genes were selected to perform a technical validation by qPCR in the same RNA samples (Fig. 4). The selection was done according to the FC and the function of the gene as described in the literature. qPCRs were performed with technical triplicates using 500 ng of total RNA for each reaction, SYBR Green supermix (Qiagen) and gene-specific primers disposed on plaques (SABiociences) in an ABI 7900HT Fast Real-Time PCR System (Applied Biosystems, Foster City, CA) according to manufacturer’s instructions. Threshold cycle (Ct) values were obtained and the expression level of each gene was normalized by the ΔCt method using the housekeeping gene B2M, whose expression was shown to present great stability across the groups, as endogenous control. Fold-changes against the respective control group were calculated as 2^−ΔΔct^.

## Electronic supplementary material

Supplementary information
