# Supplementary material for: Transcriptomic integration of D4R and MOR signaling in the rat caudate putamen
Source: Sci Rep. 2018 May 9;8:7337. doi: 10.1038/s41598-018-25604-4 (PMC5943359; doi:10.1038/s41598-018-25604-4)
Supplement: Supplementary file 1 — Supplementary information [file 41598_2018_25604_MOESM1_ESM.doc]

**Supplementary Material**

**Transcriptomic integration of D_4_R and MOR signaling in the rat caudate putamen**

Alejandra Valderrama-Carvajal, Haritz Irizar, Belén Gago, Haritz Jimenez-Urbieta, Kjell Fuxe, María C. Rodriguez-Oroz, David Otaegui, Alicia Rivera

**Supplementary Table 1. Fold Change values of the “spurious genes” in control animals.** Red labeled values indicate upregulation (FC>2) and blue labeled values mean downregulation (FC<-2).

| **Probeset ID** | **Gene Symbol** | **Entrez Gene ID** | **Avg.Expr (log2)** | **Fold-change** | **p-value** |
| --- | --- | --- | --- | --- | --- |
| 1385723_at | --- | --- | 4.18 | **-2.83** | 1.40E-06 |
| 1377445_at | --- | --- | 5.82 | **-2.61** | 3.37E-02 |
| 1367704_at | Ap2b1 /// LOC103694866 | 140670 /// 103694866 | 6.90 | **-2.59** | 2.59E-07 |
| 1376137_at | Plekhb2 | 301337 | 2.38 | **-2.57** | 4.45E-04 |
| 1368025_at | Ddit4 | 140942 | 6.49 | **-2.50** | 2.07E-03 |
| 1370652_at | Ntrk2 | 25054 | 3.52 | **-2.50** | 1.39E-05 |
| 1384717_at | --- | --- | 1.17 | **-2.50** | 8.89E-03 |
| 1376840_at | --- | --- | 1.42 | **-2.21** | 7.79E-04 |
| 1376468_at | Hars | 307492 | 4.25 | **-2.17** | 9.58E-04 |
| 1394585_at | --- | --- | 5.56 | **-2.17** | 4.07E-05 |
| 1397569_at | --- | --- | 5.26 | **-2.07** | 1.30E-02 |
| 1381147_at | Arhgap32 | 315530 | 5.89 | **-2.03** | 1.77E-04 |
| 1376362_at | Nptxr | 81005 | 5.91 | **2.01** | 4.59E-02 |
| 1390865_at | Cadps2 | 312166 | 5.26 | **2.07** | 4.92E-02 |
| 1374046_at | Hs3st2 | 293451 | 5.05 | **2.34** | 3.87E-02 |
| 1368883_at | Nov | 81526 | 4.95 | **2.37** | 3.25E-02 |
| 1387032_at | Cck | 25298 | 7.62 | **2.54** | 4.28E-02 |
| 1384093_at | C1ql3 | 680404 | 5.55 | **2.57** | 4.40E-02 |
| 1396010_at | --- | --- | 1.56 | **3.37** | 4.92E-02 |
| 1376734_at | Nov | 81526 | 5.71 | **3.54** | 1.08E-02 |
| 1390838_at | Satb2 | 501145 | 4.42 | **3.78** | 2.56E-02 |

**Supplementary Table 2. Full list of differentially expressed genes (DEGs) between groups (only with gene symbol).** Red labeled values indicate upregulation (FC>2) and blue labeled values mean downregulation (FC<-2) at p-value < 0.05 (uncorrected). Abbreviations: C, control; M, morphine; PD, PD168,077; MPD, morphine + PD168,077.

| **Probeset ID** | **Gene Symbol** | **SINGLE TREATMENT vs. CONTROLS** | | | | | | **CO-TREATMENT vs. SINGLE TREATMENT** | | | |
| --- | --- | --- | --- | --- | --- | --- | --- | --- | --- | --- | --- |
|  |  | **Morphine vs control** | | **Morphine-PD vs control** | | **PD vs control** | | **Morphine-PD vs Morphine** | | **Morphine-PD vs PD** | |
|  |  | 1h | 2h | 1h | 2h | 1h | 2h | 1h | 2h | 1h | 2h |
| 1368247_at | Hspa1a /// Hspa1b | 3.75 | **40.71** | **17.74** | **28.61** | 3.63 | 1.32 | 4.74 | -1.42 | 4.89 | **21.76** |
| 1370912_at | Hspa1a | 2.16 | **25.76** | **6.42** | **12.36** | 2.37 | 1.22 | 2.98 | -2.09 | 2.71 | **10.13** |
| 1396339_at | Fam19a1 | 2.01 | -1.51 | **5.29** | **6.09** | 1.10 | 1.47 | 2.63 | **9.20** | **4.80** | **4.15** |
| 1377934_at | Exph5 | 1.27 | -1.36 | **4.14** | **4.60** | 1.28 | 1.40 | **3.26** | **6.27** | **3.24** | **3.29** |
| 1390649_at | Slc30a3 | 1.96 | -1.45 | **4.80** | **3.60** | 1.85 | 1.30 | **2.45** | **5.23** | **2.60** | **2.78** |
| 1393708_at | Bhlhe22 | 1.61 | -1.33 | **7.91** | **4.14** | 2.25 | 1.06 | **4.90** | **5.50** | **3.52** | **3.91** |
| 1377146_at | Vip | 2.18 | -1.01 | **7.80** | **5.73** | **2.71** | 1.29 | **3.58** | **5.77** | **2.88** | **4.42** |
| 1390943_at | Rsrp1 | 1.64 | **3.57** | **2.69** | 1.70 | 1.45 | 1.11 | 1.64 | -2.10 | 1.86 | 1.53 |
| 1368574_at | Adra1b | 1.62 | -1.31 | **2.11** | **3.49** | 1.22 | -1.14 | 1.30 | **4.57** | 1.72 | **3.97** |
| 1376726_at | Car10 | 1.98 | -1.26 | **4.03** | **3.73** | 1.71 | 1.29 | 2.04 | **4.71** | **2.36** | **2.88** |
| 1369035_a_at | Kcnj6 | **3.36** | -1.26 | **7.09** | **3.80** | **2.99** | 1.43 | 2.11 | **4.77** | **2.37** | **2.66** |
| 1378153_at | --- | 1.47 | -1.40 | **2.86** | **2.98** | 1.15 | 1.31 | 1.94 | **4.16** | **2.48** | **2.27** |
| 1381206_at | Plcxd2 | 2.10 | -1.49 | **3.50** | **2.74** | 1.54 | 1.21 | 1.67 | **4.07** | **2.28** | **2.26** |
| 1388972_at | Rtn4r | 1.45 | -1.27 | **4.78** | **3.47** | 1.81 | 1.41 | **3.30** | **4.41** | **2.63** | **2.46** |
| 1378074_at | Pdk4 | 2.01 | **4.80** | 2.44 | **4.18** | 2.25 | 1.08 | 1.21 | -1.15 | 1.09 | **3.85** |
| 1380948_at | --- | 1.31 | -1.42 | **2.50** | **2.53** | 1.26 | 1.18 | 1.91 | **3.59** | 1.99 | **2.13** |
| 1386969_at | Nrn1 | 2.43 | -1.15 | **6.63** | **4.06** | **2.60** | 1.36 | **2.73** | **4.67** | 2.55 | **2.98** |
| 1370418_s_at | Syt17 | 1.74 | -1.25 | **3.77** | **3.29** | 1.53 | 1.37 | **2.17** | **4.10** | **2.47** | **2.41** |
| 1367577_at | Hspb1 | 1.12 | **3.35** | 1.33 | **2.06** | 1.42 | -1.27 | 1.20 | -1.62 | -1.06 | **2.63** |
| **Probeset ID** | **Gene Symbol** | **SINGLE TREATMENT vs. CONTROLS** | | | | | | **CO-TREATMENT vs. SINGLE TREATMENT** | | | |
|  |  | **Morphine vs control** | | **Morphine-PD vs control** | | **PD vs control** | | **Morphine-PD vs Morphine** | | **Morphine-PD vs PD** | |
|  |  | 1h | 2h | 1h | 2h | 1h | 2h | 1h | 2h | 1h | 2h |
| 1383457_at | Mfsd4 | **2.24** | -1.13 | **5.49** | **3.96** | **2.36** | 1.41 | **2.46** | **4.49** | **2.33** | **2.81** |
| 1390539_at | --- | 1.19 | -1.59 | 1.67 | **2.06** | -1.02 | 1.18 | 1.40 | **3.27** | 1.71 | 1.74 |
| 1393408_at | Neurod6 | **2.38** | -1.10 | **7.95** | **4.11** | **3.05** | 1.63 | **3.34** | **4.54** | **2.61** | **2.52** |
| 1383757_at | LOC100362176 | 1.86 | -1.20 | **2.87** | **3.34** | 1.48 | 1.63 | 1.54 | **3.99** | 1.94 | **2.05** |
| 1389362_at | Ptpn3 | 2.49 | -1.15 | **4.90** | **3.79** | 2.23 | 1.48 | 1.97 | **4.35** | 2.19 | 2.56 |
| 1398305_at | Syt17 | 1.49 | -1.16 | **3.49** | **3.52** | 1.10 | 1.50 | **2.33** | **4.07** | **3.18** | **2.35** |
| 1378809_at | Gpr165 | **2.92** | -1.01 | 1.44 | 1.29 | 1.24 | 1.29 | -2.03 | 1.30 | 1.16 | 1.00 |
| 1390777_at | Sc5d | 1.55 | **2.41** | 1.33 | 1.16 | 1.13 | 1.07 | -1.16 | **-2.08** | 1.18 | 1.09 |
| 1375771_at | Sidt1 | 1.45 | -1.28 | **3.17** | **2.97** | 1.50 | 1.06 | **2.18** | **3.80** | 2.11 | **2.80** |
| 1382527_at | Plcxd2 | 1.64 | -1.37 | **2.87** | **2.68** | 1.46 | 1.21 | 1.75 | **3.67** | 1.96 | 2.21 |
| 1369770_at | Sstr1 | **2.00** | -1.13 | **4.59** | **3.48** | **2.15** | 1.25 | **2.29** | **3.93** | **2.14** | **2.78** |
| 1376319_at | Sema3c | 1.67 | -1.33 | **2.17** | **2.37** | 1.43 | 1.30 | 1.30 | **3.16** | 1.52 | 1.83 |
| 1393795_at | Zeb2 | 1.24 | 1.73 | 1.03 | -1.50 | **2.29** | -1.05 | -1.20 | **-2.60** | **-2.22** | -1.43 |
| 1373008_x_at | Rtn4r | 1.38 | -1.40 | **3.74** | **2.32** | 1.68 | 1.15 | **2.72** | **3.24** | **2.22** | **2.01** |
| 1370214_at | Pvalb | 1.07 | -1.52 | 1.52 | 1.90 | -1.35 | 1.17 | 1.42 | **2.89** | **2.05** | 1.62 |
| 1388340_at | Ns5atp9 | -1.27 | 1.93 | -1.42 | -1.16 | 1.54 | 1.16 | -1.12 | **-2.25** | **-2.19** | -1.35 |
| 1391575_at | Hapln4 | -1.01 | -1.31 | 1.27 | **2.27** | -1.12 | 1.17 | 1.28 | **2.98** | 1.43 | 1.94 |
| 1390622_at | Cux2 | 1.70 | -1.24 | **3.72** | **2.64** | 1.21 | 1.37 | **2.19** | **3.28** | **3.08** | 1.92 |
| 1387341_a_at | Mbp | -1.11 | 1.41 | -1.47 | -1.84 | 1.09 | -1.14 | -1.32 | **-2.59** | -1.60 | -1.61 |
| 1376909_at | Rasl10a | 1.13 | -1.50 | **2.28** | 1.70 | 1.20 | 1.22 | **2.02** | **2.55** | 1.90 | 1.40 |
| 1378960_at | --- | -1.10 | **3.43** | 1.45 | 2.70 | 1.42 | 1.04 | 1.59 | -1.27 | 1.03 | 2.59 |
| 1373648_at | Mlip | 1.15 | -1.31 | **2.43** | 1.99 | 1.25 | 1.11 | **2.11** | **2.59** | 1.95 | 1.79 |
| 1377857_at | Tmem196 | 1.51 | -1.23 | 1.98 | **2.54** | 1.04 | 1.21 | 1.32 | **3.11** | 1.90 | **2.09** |
| 1370439_a_at | Kcnc2 | 1.67 | -1.17 | **3.48** | **2.83** | 1.54 | 1.47 | **2.08** | **3.29** | **2.26** | 1.93 |
| 1370428_x_at | RT1-A2 /// RT1-A3 /// RT1-EC2 | -1.23 | 1.61 | -2.07 | -1.65 | 1.53 | 1.35 | -1.68 | **-2.66** | **-3.16** | -2.23 |
| **Probeset ID** | **Gene Symbol** | **SINGLE TREATMENT vs. CONTROLS** | | | | | **CO-TREATMENT vs. SINGLE TREATMENT** | | | | |
|  |  | **Morphine vs control** | | **Morphine-PD vs control** | | **PD vs control** | | **Morphine-PD vs Morphine** | | **Morphine-PD vs PD** | |
|  |  | 1h | 2h | 1h | 2h | 1h | 2h | 1h | 2h | 1h | 2h |
| 1398457_at | Slc6a7 | 1.42 | -1.26 | **2.85** | **2.33** | 1.54 | 1.22 | **2.01** | **2.92** | 1.85 | 1.91 |
| 1376537_at | Ptpn3 | 1.56 | -1.18 | **2.25** | **2.60** | 1.33 | 1.14 | 1.44 | **3.05** | 1.69 | **2.29** |
| 1376198_at | Clmp | 1.24 | -1.41 | 1.41 | 1.74 | 1.15 | -1.02 | 1.14 | **2.45** | 1.23 | 1.77 |
| 1380057_at | --- | 1.78 | -1.35 | **3.95** | 1.98 | 1.78 | 1.68 | **2.22** | **2.68** | **2.21** | 1.18 |
| 1374168_at | Sphkap | 1.44 | -1.28 | 1.75 | 1.99 | 1.17 | 1.12 | 1.21 | **2.54** | 1.50 | 1.78 |
| 1377663_at | Rnd3 | -1.11 | 1.56 | -1.64 | -1.31 | 1.30 | -1.18 | -1.48 | **-2.04** | **-2.14** | -1.11 |
| 1375388_at | --- | 1.28 | **2.47** | 1.54 | 1.90 | 1.03 | -1.04 | 1.20 | -1.30 | 1.50 | 1.97 |
| 1390828_at | Npy1r | 1.49 | -1.25 | **2.55** | 1.86 | 1.48 | 1.21 | 1.71 | **2.32** | 1.72 | 1.53 |
| 1387360_at | Stx1a | 1.26 | -1.24 | **2.34** | **2.27** | 1.48 | 1.11 | 1.86 | **2.82** | 1.58 | **2.05** |
| 1390337_at | Kcnj9 | 1.25 | -1.26 | **2.47** | **2.04** | 1.27 | 1.24 | 1.98 | **2.58** | 1.94 | 1.64 |
| 1377279_at | LOC102554881 | 1.08 | -1.40 | 1.41 | 1.50 | 1.05 | 1.10 | 1.30 | **2.10** | 1.34 | 1.36 |
| 1368751_at | Kcns3 | 1.25 | -1.26 | **2.63** | **2.16** | 1.19 | 1.10 | **2.10** | **2.71** | **2.21** | 1.97 |
| 1374540_at | Cdca7 | -1.05 | 1.53 | -1.22 | -1.41 | 1.73 | -1.10 | -1.16 | **-2.16** | **-2.12** | -1.29 |
| 1377853_at | Tshz3 | 1.49 | -1.28 | **3.02** | 1.87 | 1.69 | -1.03 | **2.03** | **2.39** | 1.79 | 1.93 |
| 1393301_at | --- | 1.04 | -1.40 | 1.48 | 1.46 | 1.02 | -1.06 | 1.42 | **2.04** | 1.45 | 1.54 |
| 1389811_at | Rasgef1c | 1.39 | -1.06 | **2.49** | **3.06** | 1.25 | 1.03 | 1.79 | **3.24** | 1.98 | **2.96** |
| 1373991_at | Kcnj16 | 1.54 | 1.15 | -1.46 | 1.28 | -1.04 | 1.24 | **-2.25** | 1.11 | -1.40 | 1.04 |
| 1367776_at | Cdk1 | 1.02 | 1.53 | -1.01 | -1.36 | 1.62 | -1.06 | -1.03 | **-2.08** | -1.63 | -1.28 |
| 1391309_at | C1ql3 /// LOC102548876 | 1.45 | -1.13 | **2.93** | **2.69** | 1.65 | 1.15 | **2.02** | **3.04** | 1.78 | **2.34** |
| 1389408_at | LOC100359539 /// LOC103694971 /// Rrm2 | -1.02 | 1.68 | -1.15 | -1.18 | 1.75 | 1.06 | -1.13 | -1.98 | **-2.02** | -1.26 |
| 1376645_at | Medag | 1.10 | -1.30 | **2.53** | 1.71 | 1.23 | -1.09 | **2.31** | **2.23** | **2.05** | 1.85 |
| 1387497_at | Npy5r | 1.28 | -1.32 | 1.87 | 1.57 | 1.43 | 1.06 | 1.46 | **2.07** | 1.31 | 1.48 |
| 1371910_at | --- | 1.31 | -1.16 | **2.75** | **2.37** | 1.37 | 1.09 | **2.11** | **2.76** | **2.01** | **2.18** |
| 1384780_at | Cpne4 | **2.21** | -1.11 | **3.41** | **2.58** | 1.54 | 1.42 | 1.54 | **2.86** | **2.21** | 1.81 |
| **Probeset ID** | **Gene Symbol** | **SINGLE TREATMENT vs. CONTROLS** | | | | | | **CO-TREATMENT vs. SINGLE TREATMENT** | | | |
|  |  | **Morphine vs control** | | **Morphine-PD vs control** | | **PD vs control** | | **Morphine-PD vs Morphine** | | **Morphine-PD vs PD** | |
|  |  | 1h | 2h | 1h | 2h | 1h | 2h | 1h | 2h | 1h | 2h |
| 1369164_a_at | Trpc4 | **2.24** | 1.41 | 1.47 | 1.52 | 1.35 | 1.26 | -1.52 | 1.08 | 1.09 | 1.20 |
| 1370139_a_at | Trpc6 | 1.46 | -1.34 | 1.67 | 1.81 | 1.34 | 1.22 | 1.14 | **2.42** | 1.25 | 1.48 |
| 1367888_at | Cdhr1 | 1.04 | -1.41 | 1.55 | 1.48 | 1.30 | 1.47 | 1.48 | **2.09** | 1.19 | 1.01 |
| 1394135_at | --- | 1.48 | -1.11 | **2.89** | **2.51** | 1.47 | 1.25 | 1.95 | **2.79** | 1.97 | **2.00** |
| 1380218_at | Cdhr4 | 1.25 | 1.90 | -1.54 | 1.14 | 1.80 | 1.67 | -1.93 | -1.67 | **-2.78** | -1.47 |
| 1388650_at | Top2a | -1.29 | 1.60 | -1.53 | -1.08 | 1.37 | 1.11 | -1.19 | -1.72 | **-2.10** | -1.19 |
| 1397871_at | --- | 1.24 | -1.22 | **2.04** | 1.80 | 1.17 | 1.23 | 1.64 | **2.20** | 1.74 | 1.47 |
| 1372639_at | Trim54 | -1.11 | -1.26 | 1.33 | 1.93 | -1.19 | 1.17 | 1.47 | **2.44** | 1.58 | 1.66 |
| 1379867_at | Adgra1 | 1.48 | -1.21 | **2.70** | **2.13** | 1.72 | 1.23 | 1.82 | **2.58** | 1.57 | 1.74 |
| 1381955_at | Fam183b | 1.21 | 2.12 | -1.35 | 1.38 | 1.78 | 2.00 | -1.64 | -1.54 | **-2.41** | -1.45 |
| 1374176_at | Lurap1l | 1.27 | -1.30 | 1.53 | 1.55 | -1.07 | 1.03 | 1.20 | **2.03** | 1.63 | 1.51 |
| 1389698_at | Unc13b | 1.12 | -1.28 | 1.76 | 1.65 | 1.21 | 1.19 | 1.57 | **2.11** | 1.46 | 1.39 |
| 1391878_at | RGD1560020_predicted | -1.02 | 1.54 | -1.38 | -1.10 | 1.46 | 1.01 | -1.36 | -1.70 | **-2.02** | -1.11 |
| 1370995_at | Pou2f1 | 1.26 | -1.54 | -1.66 | -1.43 | 1.11 | -1.54 | **-2.10** | 1.07 | -1.84 | 1.07 |
| 1383644_at | Npr3 | 1.23 | -1.08 | 1.47 | **2.64** | 1.15 | 1.13 | 1.20 | **2.84** | 1.27 | **2.34** |
| 1379373_at | --- | 1.51 | -1.19 | 1.97 | **2.08** | 1.15 | 1.07 | 1.30 | **2.48** | 1.72 | 1.94 |
| 1398621_at | Ak7 | 1.46 | 1.55 | -1.47 | 1.07 | 1.79 | 1.71 | -2.14 | -1.45 | **-2.64** | -1.60 |
| 1396366_at | Cdh12 | 1.22 | -1.21 | 1.99 | 1.66 | 1.33 | 1.13 | 1.64 | **2.02** | 1.50 | 1.47 |
| 1379445_at | --- | **2.04** | 1.11 | 1.41 | 1.67 | 1.00 | 1.46 | -1.44 | 1.50 | 1.41 | 1.14 |
| 1376396_a_at | Tex40 | -1.11 | -1.14 | 1.72 | **2.14** | 1.18 | 1.25 | 1.90 | **2.43** | 1.46 | 1.71 |
| 1376873_at | Cbln1 | 1.48 | -1.40 | **4.02** | 1.73 | 1.70 | 1.51 | **2.72** | 2.42 | 2.37 | 1.15 |
| 1395160_at | --- | 1.16 | -1.19 | 1.61 | 1.68 | 1.13 | 1.16 | 1.39 | **2.00** | 1.43 | 1.45 |
| 1379500_at | Ccdc117 | 1.40 | **2.06** | 1.65 | 1.67 | 1.33 | 1.12 | 1.18 | -1.23 | 1.24 | 1.50 |
| 1392965_a_at | Smoc2 | 1.04 | 1.48 | -1.55 | -1.06 | 1.67 | 1.31 | -1.62 | -1.58 | **-2.59** | -1.40 |
| 1378997_at | Ephb6 | 1.33 | -1.08 | 1.86 | **2.37** | 1.49 | -1.01 | 1.39 | **2.57** | 1.25 | **2.40** |
| **Probeset ID** | **Gene Symbol** | **SINGLE TREATMENT vs. CONTROLS** | | | | | | **CO-TREATMENT vs. SINGLE TREATMENT** | | | |
|  |  | **Morphine vs control** | | **Morphine-PD vs control** | | **PD vs control** | | **Morphine-PD vs Morphine** | | **Morphine-PD vs PD** | |
|  |  | 1h | 2h | 1h | 2h | 1h | 2h | 1h | 2h | 1h | 2h |
| 1391330_at | Dnah12 | 1.25 | 1.76 | -1.48 | 1.07 | 1.78 | 1.69 | -1.85 | -1.65 | **-2.64** | -1.58 |
| 1390632_at | Rspo3 | 1.27 | -1.04 | **3.02** | **2.66** | 1.30 | 1.84 | **2.38** | **2.77** | **2.33** | 1.44 |
| 1389726_at | Lsm11 | 1.40 | -1.13 | **2.01** | **2.06** | 1.45 | 1.13 | 1.44 | **2.33** | 1.39 | 1.82 |
| 1377659_at | Mlf1 | 1.18 | 1.70 | -1.57 | -1.03 | 1.58 | 1.82 | -1.86 | -1.76 | **-2.49** | -1.88 |
| 1387383_at | Gabbr2 | 1.49 | -1.12 | **2.50** | **2.12** | 1.36 | 1.24 | 1.68 | **2.38** | 1.84 | 1.72 |
| 1381901_at | Tmem212 | 1.19 | 1.65 | -1.38 | 1.01 | 1.66 | 1.77 | -1.64 | -1.64 | **-2.29** | -1.76 |
| 1388722_at | Dnajb1 | 1.21 | **2.43** | 1.54 | **2.22** | 1.11 | 1.14 | 1.27 | -1.09 | 1.39 | 1.95 |
| 1381597_at | LOC688778 | 1.43 | -1.09 | **2.50** | **2.33** | 1.56 | 1.00 | 1.76 | **2.53** | 1.60 | **2.32** |
| 1395436_at | --- | 1.48 | -1.38 | -1.19 | -1.51 | 1.75 | -1.04 | -1.76 | -1.10 | **-2.08** | -1.46 |
| 1396567_at | --- | 1.60 | 1.16 | 1.20 | **2.10** | 1.31 | 1.03 | -1.34 | 1.80 | -1.09 | **2.04** |
| 1380425_at | Rnasel | 1.62 | -1.29 | **2.31** | 1.34 | 1.49 | 1.08 | 1.42 | 1.73 | 1.54 | 1.24 |
| 1391654_at | --- | 1.13 | **2.21** | 1.42 | 1.93 | 1.19 | 1.10 | 1.25 | -1.14 | 1.20 | 1.76 |
| 1389975_at | Elavl4 | 1.41 | -1.08 | 1.97 | **2.22** | 1.49 | 1.16 | 1.40 | **2.41** | 1.32 | 1.91 |
| 1393581_at | Aspm | -1.01 | 1.44 | -1.15 | -1.12 | 1.77 | 1.00 | -1.14 | -1.62 | **-2.03** | -1.13 |
| 1391764_at | --- | 1.38 | -1.20 | 1.93 | 1.71 | 1.12 | 1.07 | 1.39 | **2.06** | 1.72 | 1.60 |
| 1397313_at | Pnrc2 | 1.15 | 1.17 | 1.18 | -1.88 | 1.45 | 1.30 | 1.03 | **-2.21** | -1.23 | **-2.45** |
| 1390026_at | Bag3 | 1.08 | **2.10** | 1.02 | 1.84 | -1.01 | 1.06 | -1.06 | -1.14 | 1.03 | 1.74 |
| 1369067_at | Nr4a3 | **2.14** | 1.79 | 1.94 | 1.68 | **2.41** | 1.37 | -1.10 | -1.06 | -1.24 | 1.23 |
| 1383800_at | Fam155a | 1.55 | -1.18 | **2.24** | 1.70 | 1.29 | 1.17 | 1.44 | **2.01** | 1.73 | 1.45 |
| 1369908_at | Crhbp | 1.28 | -1.22 | **2.64** | 1.45 | 1.08 | 1.09 | **2.06** | 1.77 | **2.44** | 1.32 |
| 1396040_at | Shank1 | 1.31 | -1.13 | 1.89 | 1.95 | 1.35 | 1.19 | 1.44 | **2.21** | 1.40 | 1.64 |
| 1398097_at | --- | 1.19 | -1.05 | 1.70 | **2.32** | -1.00 | 1.08 | 1.44 | **2.43** | 1.71 | **2.14** |
| 1368564_at | Slc17a6 | **2.85** | -1.29 | **3.36** | 1.72 | 2.08 | 1.59 | 1.18 | 2.23 | 1.61 | 1.08 |
| 1378233_at | Sync | 1.41 | -1.08 | **2.44** | **2.07** | 1.52 | 1.06 | 1.72 | **2.23** | 1.60 | 1.95 |
| 1387288_at | Neurod1 | 1.13 | -1.02 | **2.84** | **2.44** | 1.43 | 1.33 | **2.50** | **2.50** | 1.98 | 1.83 |
| **Probeset ID** | **Gene Symbol** | **SINGLE TREATMENT vs. CONTROLS** | | | | | | **CO-TREATMENT vs. SINGLE TREATMENT** | | | |
|  |  | **Morphine vs control** | | **Morphine-PD vs control** | | **PD vs control** | | **Morphine-PD vs Morphine** | | **Morphine-PD vs PD** | |
|  |  | 1h | 2h | 1h | 2h | 1h | 2h | 1h | 2h | 1h | 2h |
| 1395474_at | --- | 1.72 | -1.31 | **3.59** | 1.55 | **2.20** | 1.31 | 2.08 | 2.03 | 1.63 | 1.18 |
| 1393440_at | --- | 1.24 | -1.11 | 1.95 | **2.02** | 1.37 | 1.28 | 1.57 | **2.23** | 1.43 | 1.57 |
| 1369646_at | Oprl1 | 1.56 | -1.21 | **2.01** | 1.47 | 1.40 | 1.03 | 1.29 | 1.78 | 1.43 | 1.43 |
| 1374747_at | Cdk14 | 1.57 | -1.07 | **2.50** | **2.18** | 1.36 | 1.32 | 1.60 | **2.33** | 1.84 | 1.65 |
| 1393386_at | Fam183b | 1.24 | 1.61 | -1.74 | 1.20 | 1.74 | 1.75 | -2.15 | -1.34 | **-3.02** | -1.46 |
| 1373559_at | --- | 1.51 | -1.08 | **2.64** | **2.05** | 1.53 | 1.15 | 1.75 | **2.21** | 1.72 | 1.79 |
| 1368120_at | Nell1 | 1.67 | -1.12 | **2.02** | 1.87 | 1.17 | 1.28 | 1.21 | **2.09** | 1.72 | 1.46 |
| 1374667_at | Fhod3 | 1.55 | -1.04 | **2.53** | **2.24** | 1.39 | 1.35 | 1.63 | **2.34** | 1.82 | 1.66 |
| 1373062_at | Sulf1 | 1.55 | -1.29 | **2.45** | 1.26 | 1.52 | 1.38 | 1.58 | 1.63 | 1.61 | -1.09 |
| 1383530_at | Nrip3 | 1.34 | -1.08 | 1.96 | 1.94 | 1.27 | 1.21 | 1.46 | **2.09** | 1.55 | 1.60 |
| 1391521_at | LOC100910774 | 1.76 | -1.07 | **2.46** | **2.08** | 1.49 | 1.25 | 1.40 | **2.22** | 1.65 | 1.66 |
| 1367648_at | Igfbp2 | 1.09 | -1.31 | 1.83 | 1.44 | -1.18 | 1.17 | 1.68 | 1.89 | **2.17** | 1.23 |
| 1382204_at | Trhde | 1.39 | -1.07 | **2.15** | **2.08** | 1.33 | 1.27 | 1.55 | **2.22** | 1.61 | 1.64 |
| 1379920_at | --- | 1.49 | -1.03 | **2.32** | **2.25** | 1.15 | 1.30 | 1.56 | **2.31** | **2.02** | 1.72 |
| 1372332_at | Mef2c | 1.07 | -1.07 | 1.79 | 1.93 | 1.12 | 1.15 | 1.68 | **2.07** | 1.60 | 1.67 |
| 1391345_at | Bmper | 1.51 | -1.04 | **2.66** | **2.21** | 1.55 | 1.21 | 1.77 | **2.30** | 1.72 | 1.82 |
| AFFX_Rat_beta-actin_5_at | --- | -1.04 | 1.06 | 1.05 | **-2.14** | 1.06 | 1.09 | 1.09 | **-2.27** | -1.01 | **-2.33** |
| 1375707_at | --- | 1.02 | 1.55 | -1.71 | 1.10 | 1.36 | 1.43 | -1.74 | -1.41 | **-2.33** | -1.30 |
| 1376500_at | Tspan17 | 1.31 | -1.08 | **2.02** | 1.93 | 1.30 | 1.20 | 1.54 | **2.08** | 1.55 | 1.60 |
| 1376829_at | Unc5d | **2.84** | -1.15 | **3.40** | 1.65 | 1.69 | 1.60 | 1.20 | 1.89 | 2.01 | 1.03 |
| 1387462_at | Chrm3 | 1.46 | -1.17 | **2.40** | 1.45 | 1.25 | 1.15 | 1.64 | 1.71 | 1.91 | 1.27 |
| 1393291_at | Ube2ql1 | 1.55 | -1.11 | **2.52** | 1.74 | 1.57 | 1.19 | 1.63 | 1.93 | 1.61 | 1.47 |
| 1378171_at | Nrp2 | 1.81 | -1.25 | **2.22** | 1.51 | 1.62 | 1.57 | 1.23 | 1.89 | 1.37 | -1.04 |
| 1389528_s_at | Jun | 1.44 | **2.10** | **2.08** | 2.00 | 1.57 | 1.22 | 1.45 | -1.05 | 1.32 | 1.64 |
| **Probeset ID** | **Gene Symbol** | **SINGLE TREATMENT vs. CONTROLS** | | | | | | **CO-TREATMENT vs. SINGLE TREATMENT** | | | |
|  |  | **Morphine vs control** | | **Morphine-PD vs control** | | **PD vs control** | | **Morphine-PD vs Morphine** | | **Morphine-PD vs PD** | |
|  |  | 1h | 2h | 1h | 2h | 1h | 2h | 1h | 2h | 1h | 2h |
| 1375420_at | Tp53i11 | 1.71 | -1.12 | **3.20** | 1.84 | 1.76 | 1.30 | 1.87 | **2.06** | 1.82 | 1.41 |
| 1375961_at | Frzb | 1.00 | -1.11 | 1.64 | 1.82 | 1.27 | 1.36 | 1.64 | **2.01** | 1.29 | 1.34 |
| 1369113_at | Grem1 | 1.24 | -1.05 | 1.93 | 1.84 | -1.06 | 1.10 | 1.56 | 1.94 | **2.04** | 1.66 |
| 1369019_at | Chrna5 | 1.38 | -1.03 | **3.14** | 2.30 | 2.39 | 1.67 | 2.27 | 2.38 | 1.31 | 1.37 |
| 1384512_at | --- | 1.68 | -1.10 | **2.20** | 1.77 | 1.70 | 1.25 | 1.31 | 1.95 | 1.30 | 1.42 |
| 1379414_at | Ube2ql1 | 1.52 | -1.00 | **2.46** | **2.28** | 1.42 | 1.21 | 1.62 | **2.28** | 1.73 | 1.88 |
| 1391019_at | Slitrk1 | 1.64 | -1.08 | **2.17** | 1.71 | 1.40 | 1.13 | 1.32 | 1.85 | 1.55 | 1.51 |
| 1367679_at | Cd74 | 1.57 | 1.31 | 1.64 | -1.11 | **2.11** | -1.17 | 1.05 | -1.45 | -1.28 | 1.06 |
| 1384389_at | Rtn4rl2 | 1.47 | -1.02 | **2.88** | **2.16** | 1.61 | 1.41 | 1.96 | **2.19** | 1.80 | 1.53 |
| 1390146_at | Tmem150c | 1.36 | -1.06 | 1.80 | 1.90 | 1.30 | 1.41 | 1.32 | **2.02** | 1.38 | 1.35 |
| 1368662_at | Rnf39 | 1.46 | -1.01 | **2.30** | **2.18** | 1.70 | 1.22 | 1.58 | **2.20** | 1.36 | 1.79 |
| 1368162_at | Cst6 | 1.59 | -1.09 | **2.01** | 1.64 | 1.22 | 1.25 | 1.26 | 1.78 | 1.64 | 1.31 |
| 1387337_at | Cort | 1.16 | -1.00 | **2.26** | **2.18** | 1.34 | 1.27 | 1.94 | **2.18** | 1.68 | 1.72 |
| 1396744_at | LOC100909913 /// Ndp | -1.09 | 1.04 | -1.06 | -1.95 | -1.04 | 1.04 | 1.04 | **-2.04** | -1.01 | **-2.03** |
| 1398521_at | --- | 1.15 | -1.01 | 1.81 | **2.05** | 1.19 | 1.00 | 1.58 | **2.07** | 1.52 | **2.04** |
| 1391919_at | Tcerg1l | 1.37 | -1.04 | 1.53 | 1.94 | 1.04 | -1.10 | 1.12 | **2.02** | 1.47 | **2.12** |
| 1391598_at | --- | **2.14** | 1.63 | **2.59** | 1.26 | 1.19 | 1.57 | 1.21 | -1.30 | **2.17** | -1.25 |
| 1378472_at | Unc5d | **2.40** | -1.14 | **2.60** | 1.35 | 1.67 | 1.08 | 1.08 | 1.54 | 1.55 | 1.26 |
| 1392064_at | Dlx1 | 1.23 | 1.24 | -1.04 | -1.23 | 1.94 | 1.07 | -1.29 | -1.52 | **-2.02** | -1.31 |
| 1387641_at | Rab5a | 1.31 | 1.00 | 1.42 | **-2.08** | 1.30 | 1.11 | 1.08 | **-2.08** | 1.09 | **-2.30** |
| 1387696_a_at | Glra2 | 1.61 | -1.14 | **2.25** | 1.24 | 1.44 | 1.38 | 1.40 | 1.41 | 1.56 | -1.11 |
| 1393416_at | Rasal1 | 1.37 | -1.02 | **2.63** | 1.88 | 1.52 | 1.34 | 1.91 | 1.92 | 1.73 | 1.40 |
| 1376956_at | --- | -1.01 | 1.13 | 1.92 | **2.15** | 1.10 | 1.23 | 1.93 | 1.90 | 1.75 | 1.75 |
| 1376928_at | Adamts2 | 1.12 | -1.05 | **2.34** | 1.80 | 1.57 | 1.51 | **2.10** | 1.89 | 1.50 | 1.19 |
| 1373159_at | Clstn2 | 1.35 | -1.02 | **2.08** | 1.85 | 1.25 | 1.22 | 1.54 | 1.88 | 1.66 | 1.52 |
| **Probeset ID** | **Gene Symbol** | **SINGLE TREATMENT vs. CONTROLS** | | | | | | **CO-TREATMENT vs. SINGLE TREATMENT** | | | |
|  |  | **Morphine vs control** | | **Morphine-PD vs control** | | **PD vs control** | | **Morphine-PD vs Morphine** | | **Morphine-PD vs PD** | |
|  |  | 1h | 2h | 1h | 2h | 1h | 2h | 1h | 2h | 1h | 2h |
| 1371363_at | Gpd1 | -1.15 | 1.85 | -1.13 | 1.80 | -1.22 | -1.11 | 1.02 | -1.03 | 1.08 | **2.00** |
| 1393927_at | Wnt2 | 1.39 | -1.00 | **2.56** | 1.94 | 1.51 | 1.22 | 1.84 | 1.94 | 1.69 | 1.58 |
| 1380757_at | --- | -1.51 | 1.18 | **-2.08** | -1.16 | -1.33 | -1.18 | -1.37 | -1.37 | -1.55 | 1.02 |
| 1395287_at | --- | 1.39 | -1.07 | **3.42** | 1.75 | 2.07 | -1.26 | **2.47** | 1.87 | 1.65 | **2.20** |
| 1378340_at | --- | 1.31 | 1.45 | -1.01 | 1.29 | 1.85 | **2.02** | -1.32 | -1.13 | -1.87 | -1.57 |
| 1394881_at | Fam81a | 1.34 | -1.06 | **2.20** | 1.53 | 1.41 | 1.04 | 1.64 | 1.62 | 1.56 | 1.48 |
| 1398350_at | --- | 1.44 | -1.01 | **2.02** | 1.78 | 1.52 | 1.20 | 1.40 | 1.80 | 1.33 | 1.49 |
| 1395183_at | --- | 1.43 | -1.02 | **2.95** | 1.80 | 1.65 | 1.12 | **2.06** | 1.84 | 1.79 | 1.61 |
| 1389306_at | Matn2 | 1.92 | -1.03 | **2.76** | 1.79 | 1.43 | 1.10 | 1.44 | 1.84 | 1.93 | 1.62 |
| 1382995_at | Nrp2 | **2.13** | -1.17 | **2.42** | 1.41 | 1.64 | 1.41 | 1.13 | 1.64 | 1.47 | 1.00 |
| 1374775_at | Mki67 | 1.03 | 1.20 | -1.11 | -1.15 | **2.01** | -1.10 | -1.15 | -1.39 | **-2.24** | -1.05 |
| 1367564_at | Nppa | 1.47 | -1.03 | **2.05** | 1.67 | 1.34 | 1.28 | 1.40 | 1.71 | 1.53 | 1.30 |
| 1370883_at | RT1-Da | 1.32 | 1.21 | 1.53 | -1.15 | **2.02** | -1.22 | 1.16 | -1.40 | -1.33 | 1.06 |
| 1381927_at | Pifo | 1.28 | 1.49 | -1.07 | 1.25 | **2.01** | 1.93 | -1.37 | -1.19 | **-2.15** | -1.54 |
| 1387306_a_at | Egr2 | 1.72 | 1.21 | 1.72 | 1.42 | **2.04** | 1.16 | -1.00 | 1.17 | -1.18 | 1.22 |
| 1395672_at | --- | 1.70 | -1.01 | **2.24** | 1.63 | 1.52 | 1.26 | 1.31 | 1.65 | 1.48 | 1.29 |
| 1369202_at | Mx2 | -1.18 | 1.65 | **-2.33** | 1.57 | -1.27 | -1.03 | -1.98 | -1.05 | -1.83 | 1.62 |
| 1385463_at | LOC102550863 | 1.23 | -1.04 | **2.11** | 1.52 | 1.31 | 1.15 | 1.71 | 1.58 | 1.60 | 1.32 |
| 1381080_at | Dgkg | 1.74 | -1.07 | **2.17** | 1.41 | 1.46 | 1.34 | 1.25 | 1.50 | 1.48 | 1.05 |
| 1397225_at | --- | -1.07 | 1.76 | 1.26 | **2.40** | -1.05 | 1.05 | 1.35 | 1.36 | 1.32 | **2.28** |
| 1382626_at | --- | 1.95 | -1.06 | **2.62** | 1.47 | 1.43 | 1.63 | 1.35 | 1.56 | 1.83 | -1.11 |
| 1386152_at | --- | 2.57 | -1.33 | **9.03** | 1.22 | 2.59 | 1.48 | 3.52 | 1.61 | 3.49 | -1.21 |
| 1397513_at | Ralyl | 1.87 | -1.04 | **2.17** | 1.31 | 1.28 | 1.38 | 1.16 | 1.36 | 1.70 | -1.05 |
| 1396238_at | Galnt14 | 1.34 | -1.03 | **2.12** | 1.31 | 1.32 | 1.23 | 1.58 | 1.35 | 1.61 | 1.06 |
| 1387066_a_at | Rgs12 | 1.26 | -1.13 | **2.05** | 1.12 | 1.56 | 1.27 | 1.62 | 1.27 | 1.31 | -1.13 |
| **Probeset ID** | **Gene Symbol** | **SINGLE TREATMENT vs. CONTROLS** | | | | | | **CO-TREATMENT vs. SINGLE TREATMENT** | | | |
|  |  | **Morphine vs control** | | **Morphine-PD vs control** | | **PD vs control** | | **Morphine-PD vs Morphine** | | **Morphine-PD vs PD** | |
|  |  | 1h | 2h | 1h | 2h | 1h | 2h | 1h | 2h | 1h | 2h |
| 1371113_a_at | Tfrc | -1.27 | -1.23 | -1.22 | **-2.04** | -1.28 | 1.04 | 1.03 | -1.66 | 1.05 | **-2.11** |
| 1380306_at | --- | -1.01 | **2.42** | 1.30 | **3.96** | -1.14 | -1.04 | 1.31 | 1.64 | 1.49 | **4.12** |
| 1378421_at | LOC100910996 | 1.38 | -1.05 | **2.12** | 1.18 | 1.27 | 1.35 | 1.53 | 1.24 | 1.67 | -1.15 |
| 1394957_at | Nisch | -1.10 | -1.19 | 1.04 | -1.92 | -1.06 | 1.07 | 1.15 | -1.62 | 1.11 | **-2.05** |
| 1378374_at | Fam216b | 1.02 | 1.16 | -1.13 | 1.03 | 1.86 | 1.41 | -1.16 | -1.13 | **-2.11** | -1.37 |
| 1367768_at | Lxn | 1.41 | 1.08 | **2.51** | 1.07 | 1.57 | 1.30 | 1.78 | -1.01 | 1.60 | -1.22 |
| 1378362_at | C1ql3 | 2.19 | 1.08 | **8.06** | **5.14** | **3.06** | 1.20 | **3.69** | **4.78** | **2.64** | **4.28** |
| 1386035_at | --- | 1.51 | 1.01 | **4.63** | **4.36** | 1.94 | 1.48 | **3.06** | **4.29** | 2.39 | **2.95** |
| 1395052_at | --- | 2.20 | 1.06 | **6.49** | **4.29** | **2.37** | 1.87 | **2.95** | **4.07** | **2.74** | 2.29 |
| 1368677_at | Bdnf | 1.98 | 1.07 | **5.77** | **4.12** | **2.58** | 1.78 | **2.92** | **3.86** | **2.24** | **2.32** |
| 1374280_at | Cbln2 | **2.14** | 1.05 | **4.03** | **3.62** | 1.96 | 1.56 | 1.88 | **3.46** | 2.06 | **2.31** |
| 1368987_at | Slc17a7 | 1.64 | 1.01 | **4.97** | **3.18** | 2.16 | 1.58 | **3.03** | **3.15** | 2.30 | 2.02 |
| 1369008_a_at | Olfm1 | 1.84 | 1.03 | **3.76** | **2.78** | **2.08** | 1.19 | **2.05** | **2.71** | 1.81 | **2.34** |
| 1368854_at | Vsnl1 | **2.20** | 1.03 | **2.86** | **2.69** | 1.70 | 1.36 | 1.30 | **2.62** | 1.68 | 1.98 |
| 1393614_at | E2f1 /// Necab3 | 1.64 | 1.01 | **2.87** | **2.53** | 1.85 | 1.22 | 1.75 | **2.52** | 1.55 | **2.08** |
| 1375043_at | --- | **2.28** | **3.32** | **3.30** | **4.05** | **2.12** | **2.17** | 1.45 | 1.22 | 1.56 | 1.87 |
| 1378350_at | LOC102547703 | 1.17 | 1.01 | **2.16** | **2.40** | 1.17 | 1.28 | 1.84 | **2.38** | 1.85 | 1.87 |
| 1368256_at | Serpini1 | 1.42 | 1.01 | **2.28** | **2.38** | 1.38 | 1.38 | 1.60 | **2.36** | 1.65 | 1.72 |
| 1376106_at | Tmem178a | **2.08** | 1.12 | **4.55** | **2.98** | **2.54** | 1.30 | **2.19** | **2.65** | 1.79 | **2.29** |
| 1387927_a_at | Olfm1 | 1.39 | 1.03 | **2.73** | **2.50** | 1.60 | 1.17 | 1.97 | **2.42** | 1.71 | **2.14** |
| 1368611_at | Grp | 1.10 | 1.41 | **2.34** | **4.50** | 1.33 | -1.07 | 2.13 | **3.19** | 1.76 | **4.80** |
| 1387131_at | Serpini1 | 1.61 | 1.01 | **2.97** | **2.30** | 1.70 | 1.46 | 1.84 | **2.28** | 1.75 | 1.58 |
| 1390958_at | Sertm1 | 1.47 | 1.04 | **2.85** | **2.45** | 1.56 | 1.56 | 1.93 | **2.36** | 1.83 | 1.57 |
| 1382457_at | LOC103692931 | 1.46 | 1.03 | **2.69** | **2.29** | 1.78 | 1.16 | 1.84 | **2.22** | 1.51 | 1.98 |
| 1370997_at | Homer1 | 1.38 | **2.09** | **2.13** | **2.10** | 1.64 | 1.32 | 1.55 | 1.00 | 1.30 | 1.59 |
| **Probeset ID** | **Gene Symbol** | **SINGLE TREATMENT vs. CONTROLS** | | | | | | **CO-TREATMENT vs. SINGLE TREATMENT** | | | |
|  |  | **Morphine vs control** | | **Morphine-PD vs control** | | **PD vs control** | | **Morphine-PD vs Morphine** | | **Morphine-PD vs PD** | |
|  |  | 1h | 2h | 1h | 2h | 1h | 2h | 1h | 2h | 1h | 2h |
| 1384197_at | Dlgap1 | 1.28 | 1.01 | 1.97 | **2.09** | 1.14 | 1.34 | 1.54 | **2.08** | 1.73 | 1.57 |
| 1386890_at | S100a10 | 1.39 | 1.07 | **2.64** | **2.44** | 1.65 | 1.18 | 1.90 | **2.28** | 1.60 | **2.06** |
| 1384862_at | --- | 1.83 | 1.05 | **2.53** | **2.30** | 1.46 | 1.54 | 1.38 | **2.20** | 1.73 | 1.49 |
| 1387281_a_at | Pnck | **2.27** | 1.03 | **3.32** | **2.26** | 1.42 | 1.32 | 1.46 | 2.20 | **2.34** | 1.71 |
| 1377309_at | --- | 1.75 | 1.15 | **2.25** | **2.83** | 1.36 | 1.57 | 1.29 | **2.46** | 1.66 | 1.81 |
| 1395918_at | Aldh3b2 | 1.31 | 1.07 | **2.12** | **2.40** | 1.31 | 1.41 | 1.61 | **2.24** | 1.62 | 1.70 |
| 1381595_at | --- | 1.36 | 1.03 | 1.96 | **2.14** | 1.35 | 1.08 | 1.44 | **2.09** | 1.44 | 1.99 |
| 1378298_at | Igsf21 | 1.60 | 1.04 | **3.08** | **2.18** | 1.94 | 1.41 | 1.93 | **2.11** | 1.59 | 1.54 |
| 1382651_at | --- | 1.83 | 1.07 | **2.60** | **2.36** | 1.62 | 1.38 | 1.42 | **2.20** | 1.61 | 1.71 |
| 1390317_at | RGD1561849 | 1.60 | 1.09 | **3.25** | **2.42** | **2.15** | 1.25 | **2.03** | **2.22** | 1.51 | 1.93 |
| 1387260_at | Klf4 | 1.47 | 1.94 | **2.18** | 1.96 | 1.51 | 1.32 | 1.48 | 1.01 | 1.44 | 1.49 |
| 1383931_at | Ptprk | 1.50 | 1.01 | **2.39** | 1.99 | 1.33 | 1.14 | 1.59 | 1.96 | 1.79 | 1.74 |
| 1389514_at | Lingo1 | 1.36 | 1.02 | **2.17** | 1.99 | 1.43 | 1.13 | 1.59 | 1.95 | 1.52 | 1.76 |
| 1382358_at | Sox5 | 1.04 | 1.08 | **2.09** | 1.60 | 1.52 | 1.15 | **2.01** | 1.48 | 1.37 | 1.39 |
| 1396023_at | Dpp10 | 1.53 | 1.05 | 1.75 | **2.12** | 1.29 | 1.18 | 1.14 | **2.02** | 1.36 | 1.81 |
| 1369167_at | Gfra2 | 1.45 | 1.06 | 1.81 | **2.15** | 1.24 | 1.20 | 1.25 | **2.03** | 1.45 | 1.79 |
| 1382280_at | Uqcrc2 | 1.14 | -1.00 | 1.24 | -1.89 | 1.13 | 1.10 | 1.09 | -1.89 | 1.09 | **-2.08** |
| 1385130_at | Fezf2 | 1.09 | 1.28 | **2.35** | **2.27** | 1.70 | 1.19 | **2.15** | 1.77 | 1.38 | 1.91 |
| 1393129_at | P4ha3 | 1.41 | 1.10 | **3.22** | **2.30** | 1.42 | 1.06 | **2.28** | **2.10** | **2.27** | **2.18** |
| 1386041_a_at | Klf2 | 1.18 | 1.87 | **2.10** | 1.90 | 1.24 | 1.09 | 1.78 | 1.02 | 1.69 | 1.74 |
| 1367970_at | Pfn2 | 1.10 | -1.09 | 1.32 | **-2.18** | 1.06 | 1.06 | 1.21 | -2.00 | 1.24 | **-2.31** |
| 1368853_at | Vsnl1 | **2.56** | 1.08 | **3.65** | 2.19 | 1.84 | 1.46 | 1.43 | 2.03 | 1.98 | 1.50 |
| 1393565_at | RGD1563441 | 1.03 | -1.09 | 1.06 | **-2.13** | -1.04 | 1.01 | 1.03 | -1.96 | 1.09 | **-2.16** |
| 1379387_at | Rgs18 | 1.42 | 1.20 | **3.66** | 1.55 | 1.85 | 1.51 | **2.57** | 1.30 | 1.98 | 1.03 |
| 1378629_at | Satb1 | 1.34 | 1.08 | 1.82 | **2.06** | 1.39 | 1.12 | 1.36 | 1.91 | 1.31 | 1.84 |
| **Probeset ID** | **Gene Symbol** | **SINGLE TREATMENT vs. CONTROLS** | | | | | | **CO-TREATMENT vs. SINGLE TREATMENT** | | | |
|  |  | **Morphine vs control** | | **Morphine-PD vs control** | | **PD vs control** | | **Morphine-PD vs Morphine** | | **Morphine-PD vs PD** | |
|  |  | 1h | 2h | 1h | 2h | 1h | 2h | 1h | 2h | 1h | 2h |
| 1371007_at | Epha5 | 1.43 | 1.03 | **2.26** | 1.81 | 1.26 | 1.21 | 1.59 | 1.76 | 1.80 | 1.50 |
| 1372539_at | Parm1 | 1.17 | 1.08 | 1.49 | **2.09** | 1.08 | 1.10 | 1.27 | 1.93 | 1.38 | 1.90 |
| 1369847_at | Kcnab1 | 1.08 | -1.05 | 1.10 | -1.90 | -1.15 | 1.06 | 1.01 | -1.81 | 1.26 | **-2.01** |
| 1381557_at | Gna14 | 1.17 | 1.08 | 1.72 | **2.18** | 1.24 | 1.00 | 1.47 | **2.02** | 1.38 | **2.18** |
| 1372953_at | Ncald | 1.40 | 1.05 | **2.01** | 1.86 | 1.46 | 1.23 | 1.43 | 1.77 | 1.38 | 1.52 |
| 1390645_at | --- | **2.06** | 1.02 | **2.75** | 1.68 | 1.51 | 1.37 | 1.34 | 1.64 | 1.82 | 1.22 |
| 1377994_at | Pmaip1 | 1.14 | 1.97 | 1.36 | **2.13** | 1.14 | 1.41 | 1.19 | 1.08 | 1.19 | 1.51 |
| 1377986_at | Scoc | 1.22 | -1.06 | 1.34 | -1.92 | 1.20 | 1.13 | 1.10 | -1.80 | 1.12 | **-2.17** |
| 1385442_at | --- | 1.65 | 1.07 | **2.58** | 1.92 | 1.97 | 1.22 | 1.56 | 1.80 | 1.31 | 1.57 |
| 1394218_s_at | Zfpm2 | 1.35 | 1.12 | 1.64 | **2.38** | 1.47 | 1.27 | 1.21 | **2.11** | 1.11 | 1.86 |
| 1383322_at | Rasl11b | 1.25 | 1.11 | **2.43** | **2.19** | 1.36 | 1.29 | 1.94 | 1.96 | 1.78 | 1.70 |
| 1390672_at | Rprm | 2.94 | 1.36 | **5.92** | **3.14** | **3.19** | 1.30 | 2.02 | 2.31 | 1.86 | 2.42 |
| 1395904_at | --- | -1.35 | -1.09 | -1.66 | **-2.05** | -1.30 | -1.13 | -1.23 | -1.88 | -1.28 | -1.81 |
| 1397754_at | Dkk3 | 1.50 | 1.20 | **2.06** | **2.60** | 1.65 | 1.30 | 1.38 | **2.17** | 1.24 | **2.00** |
| 1370454_at | Homer1 | 1.72 | 1.30 | 1.83 | 1.58 | **2.06** | 1.13 | 1.07 | 1.21 | -1.13 | 1.39 |
| 1393299_at | Dpp10 | 1.67 | 1.05 | **2.06** | 1.71 | 1.43 | 1.04 | 1.23 | 1.64 | 1.44 | 1.65 |
| 1376569_at | Klf2 | 1.34 | 1.96 | **2.02** | **2.35** | 1.34 | 1.26 | 1.50 | 1.20 | 1.50 | 1.87 |
| 1387007_at | Gfra1 | **2.05** | 1.02 | **2.51** | 1.15 | 1.64 | 1.45 | 1.23 | 1.12 | 1.53 | -1.26 |
| 1391637_at | Arhgap15 | 1.09 | 1.21 | 1.86 | **2.44** | 1.02 | 1.27 | 1.71 | **2.02** | 1.82 | 1.92 |
| 1398219_at | LOC690918 | 1.72 | 1.04 | **2.87** | 1.64 | 1.24 | 1.24 | 1.67 | 1.58 | **2.31** | 1.33 |
| 1368696_at | Fxyd7 | 1.30 | 1.16 | **2.83** | **2.17** | 1.73 | 1.31 | **2.18** | 1.88 | 1.64 | 1.66 |
| 1384392_at | Cyp26b1 | 1.85 | 1.70 | **3.87** | **4.56** | **2.30** | 1.75 | 2.09 | **2.68** | 1.68 | **2.61** |
| 1396087_at | --- | 1.17 | 1.13 | 1.86 | **2.02** | 1.41 | 1.41 | 1.59 | 1.79 | 1.32 | 1.43 |
| 1385744_at | Thsd7b | 1.74 | 1.05 | **3.48** | 1.56 | **2.22** | 1.37 | **2.00** | 1.50 | 1.57 | 1.14 |
| 1368146_at | Dusp1 | 1.27 | 1.78 | **2.05** | **2.08** | 1.31 | 1.21 | 1.61 | 1.17 | 1.56 | 1.72 |
| **Probeset ID** | **Gene Symbol** | **SINGLE TREATMENT vs. CONTROLS** | | | | | | **CO-TREATMENT vs. SINGLE TREATMENT** | | | |
|  |  | **Morphine vs control** | | **Morphine-PD vs control** | | **PD vs control** | | **Morphine-PD vs Morphine** | | **Morphine-PD vs PD** | |
|  |  | 1h | 2h | 1h | 2h | 1h | 2h | 1h | 2h | 1h | 2h |
| 1370517_at | Nptx1 | 1.99 | 1.29 | **4.01** | **2.46** | **2.75** | 1.34 | 2.02 | 1.92 | 1.46 | 1.84 |
| 1386660_at | Gng2 | **2.16** | 1.08 | **3.66** | 1.53 | **2.10** | 1.52 | 1.69 | 1.42 | 1.74 | 1.01 |
| 1392613_at | --- | 1.07 | 1.99 | 1.51 | **3.78** | 1.05 | 1.54 | 1.40 | 1.90 | 1.43 | **2.45** |
| 1384779_at | Cplx3 | 2.79 | 1.44 | **5.76** | 1.88 | 3.12 | 1.67 | 2.06 | 1.31 | 1.85 | 1.12 |
| 1374818_at | Nxph3 | 1.92 | 1.12 | **4.30** | 1.73 | **2.91** | 1.28 | **2.24** | 1.55 | 1.48 | 1.36 |
| 1368782_at | Sstr2 | 1.52 | 1.14 | **2.41** | 1.73 | 1.69 | 1.53 | 1.58 | 1.52 | 1.43 | 1.13 |
| 1370669_a_at | Pde10a | -1.21 | -1.27 | -1.37 | **-2.37** | -1.23 | -1.14 | -1.13 | -1.87 | -1.12 | **-2.08** |
| 1376911_at | Atp2b4 | **2.02** | 1.19 | **3.09** | 1.88 | **2.05** | 1.29 | 1.53 | 1.58 | 1.51 | 1.46 |
| 1390403_at | Fam43a /// LOC100909474 | 1.38 | 1.60 | 1.52 | **2.26** | 1.40 | 1.12 | 1.10 | 1.41 | 1.09 | **2.01** |
| 1379859_at | --- | -1.12 | -1.39 | -1.59 | -1.70 | -1.25 | 1.54 | -1.41 | -1.22 | -1.27 | **-2.61** |
| 1377513_at | --- | 1.84 | 1.10 | **2.82** | 1.49 | 1.81 | 1.45 | 1.54 | 1.35 | 1.56 | 1.02 |
| 1370328_at | Dkk3 | 1.40 | 1.30 | **2.12** | **2.29** | 1.53 | 1.28 | 1.51 | 1.76 | 1.38 | 1.79 |
| 1370248_at | Fxyd6 | 1.35 | 1.17 | **2.11** | 1.69 | 1.86 | 1.09 | 1.57 | 1.45 | 1.14 | 1.55 |
| 1396206_at | Dok5 | 1.37 | 1.16 | **2.41** | 1.31 | 1.39 | 1.41 | 1.76 | 1.13 | 1.74 | -1.08 |
| 1390869_at | Cbln1 | 1.47 | 1.04 | **2.45** | 1.32 | 1.27 | 1.47 | 1.66 | 1.27 | 1.93 | -1.11 |
| 1387410_at | Nr4a2 | 2.30 | 1.08 | **5.03** | 1.48 | 2.37 | 1.41 | 2.19 | 1.38 | 2.13 | 1.05 |
| 1369007_at | Nr4a2 | 3.23 | 1.08 | **10.41** | 1.32 | **4.86** | 1.71 | 3.22 | 1.22 | 2.14 | -1.30 |
| 1392110_at | --- | -1.13 | -1.27 | -1.28 | -1.71 | -1.26 | 1.24 | -1.13 | -1.35 | -1.02 | **-2.13** |


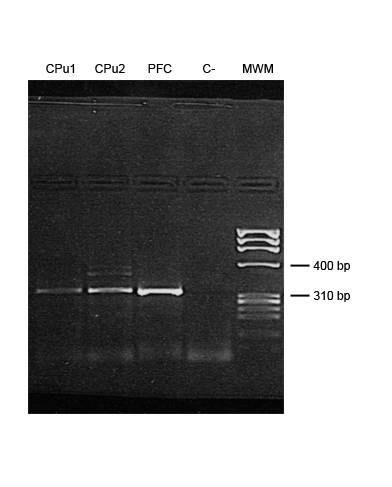


**Supplementary Figure 1. Full-length gel showing amplified PCR products (3% agarose gel electrophoresis).** Abbreviations: CPu, caudate putamen; PFC, prefrontal cortex, C-, negative control; MWM, molecular weight markers.
